# The Case for Expanding Visual Assessments During Spaceflight

**DOI:** 10.1017/S1049023X23005964

**Published:** 2023-08

**Authors:** Ethan Waisberg, Joshua Ong, Mouayad Masalkhi, Nasif Zaman, Sharif Amit Kamran, Prithul Sarker, Alireza Tavakkoli, Andrew G. Lee

**Affiliations:** 1. University College Dublin School of Medicine, Belfield, Dublin, Ireland; 2. Michigan Medicine, University of Michigan, Ann Arbor, Michigan USA; 3. Human-Machine Perception Laboratory, Department of Computer Science and Engineering, University of Nevada, Reno, Reno, Nevada USA; 4. Center for Space Medicine, Baylor College of Medicine, Houston, Texas USA; 5. Department of Ophthalmology, Blanton Eye Institute, Houston Methodist Hospital, Houston, Texas USA; 6. The Houston Methodist Research Institute, Houston Methodist Hospital, Houston, Texas USA; 7. Departments of Ophthalmology, Neurology, and Neurosurgery, Weill Cornell Medicine, New York, New York USA; 8. Department of Ophthalmology, University of Texas Medical Branch, Galveston, Texas USA; 9. University of Texas MD Anderson Cancer Center, Houston, Texas USA; 10. Texas A&M College of Medicine, Texas USA; 11. Department of Ophthalmology, The University of Iowa Hospitals and Clinics, Iowa City, Iowa USA

**Keywords:** head-mounted technology, SANS, spaceflight associated neuro-ocular syndrome, visual assessments

## Abstract

Spaceflight associated neuro-ocular syndrome (SANS) is one of the potential barriers to human long-duration spaceflight (LDSF), including a manned mission to Mars. While a large barrier, the pathophysiology of SANS is not well understood, and functional and structural findings from SANS continue to be further characterized. Currently on the International Space Station (ISS), scheduled visual assessments are static visual acuity, Amsler grid, and a self-reported survey. Additional visual assessments may help the understanding of this neuro-ophthalmic phenomenon, as well as the effects of spaceflight of overall ocular health. In this paper, a case is made for expanding scheduled visual assessments to include dynamic visual, contrast sensitivity (CS), visual field testing, and virtual reality-based metamorphopsia assessment during spaceflight. These further assessments may play a key role in helping to determine the structural and functional changes associated with SANS, which are crucial to maintain astronaut vision during LDSF, as well as for developing countermeasures. Finally, a brief discussion is provided about current challenges to expanding visual testing during spaceflight and potential solutions to these barriers, specifically head-mounted visual assessment technology.

## Introduction

Spaceflight associated neuro-ocular syndrome (SANS) is a collection of neuro-ocular imaging and clinical findings, including unilateral and bilateral optic disc edema (ODE), posterior globe flattening, hyperopic refractive error shifts, cotton wool spots, and chorioretinal folds.^1^ The visual and structural impact of SANS occurs in astronauts engaged in longer duration space missions. While a considerable barrier to future spaceflight, the pathophysiology of SANS is poorly defined. In addition, the functional and structural effects of microgravity on the neuro-ophthalmic system continue to be further characterized.

As these neuro-ocular stresses are not experienced by crews in isolation, it is crucial to consider how they together affect human physiology. The space exposome reflects a person’s total exposure to their surroundings as well as how these exposures interact with other personal characteristics like age, sex, and genetics. It is thus a unifying framework that depicts the interplay of all environmental effects on the human body. This framework will influence how space travel will affect the human system.

Currently in-orbit onboard the International Space Station (ISS), the only forms of functional visual assessment performed include static visual acuity, Amsler grid, and a self-reported survey.^2^ For these assessments, crewmembers who spend six months in-orbit are evaluated pre-launch, after 30 launch days (LD30), after LD90, and 30 days before returning to Earth. In addition, evaluations may be performed when clinically indicated. For crewmembers who spend one year in-orbit, astronauts on the ISS undergo these assessments at LD30, LD90, LD180, and LD270, in addition to 30 days before returning to Earth.^2^ Contrast sensitivity (CS) is available onboard the ISS, but only utilized when clinically indicated.^2^ However, scheduled CS assessments during spaceflight may help further characterize SANS and its pathogenesis regarding cephalad fluid shifts and intracranial pressure (ICP).

On the ISS, prior work has demonstrated that 69% of the United States crew members have at least one eye with a > 20µm increase in peripapillary retinal nerve fiber layer thickness (RNFL) on optical coherence tomography (OCT) measurements. This increased RNFL correlates with the ophthalmoscopic sign of ODE. Alongside ODE, other ocular findings in astronauts on long- or short-duration flights (around six months) include refractive changes or “hyperopic shift” and chorioretinal folds.^3^ Potentially, ODE can result in enlargement of the blind spot and other visual field scotomas, but there is currently no automated perimeter onboard the ISS. According to the SANS Evidence Report, visual distortions or decreased visual acuity that cannot be treated by glasses or contact lenses is a theoretic risk of SANS. Globe flattening (shortening of the eye’s axial length) occurs in around 16% and 29% of crewmembers during short- and long-duration space voyages, respectively, and leads to the refractive hyperopic shift in SANS.^3^ Special “Space Anticipation Glasses” with customizable positive lens power settings have been used on the ISS prophylactically to minimize such refractive errors.^1^ Currently, however, astronauts bring their own reading add spectacles on the ISS. The clinical findings in astronauts following long-duration spaceflight (LDSF) that include ODE and chorioretinal folds have been investigated using imaging technology such OCT and magnetic resonance imaging (MRI).

In this paper, a case is made for expanding scheduled visual assessments during spaceflight to include CS, dynamic visual acuity (DVA), visual field perimetry, and head-mounted metamorphopsia assessment. These clinically insightful assessments may play a key role in helping to determine the structural and functional changes associated with SANS, which are crucial to maintain astronaut vision during LDSF, as well as for developing countermeasures.

### Dynamic Visual Acuity (DVA)

Dynamic visual acuity is the ability to visualize an object in motion and is essential in a rapidly changing environment such as during spaceflight.^4^ Following 24 hours after return from LDSF, it was previously reported that astronauts had a significantly decreased DVA of 0.75 eye chart lines, with some astronauts performing similarly to individuals with vestibular impairments.^5^ As these results occurred 24 hours after landing, the decrease in DVA during and directly following a gravitational (G)-transition event is likely much greater and may pose a significant risk to astronaut mission performance during this critical period.^6^ Current research is focused on utilizing head-mounted DVA assessments to evaluate astronaut safety following G-transitions.^4^ Further DVA assessments of astronauts in-flight and post-flight are required to further understand these G-transition effects.

Advances in developing this technology is particularly useful as one of the biggest clinical issues affecting crew functions during G-transitions is motion sickness.^7^ In-flight Space Motion Sickness (SMS) has been a major topic of discussion and research at National Aeronautics and Space Administration (NASA; Washington, DC USA) recently, since most missions performed are short in duration (around ten days). But LDSF increases the risk of developing re-entry SMS. Onset of in-flight and re-entry motion sickness occurs typically within a few minutes of G-transition, with head movement sensitivity lasting for days. Notably, sudden or triggering head movements, visual reorientation illusions can cause vomiting.^8^

### Contrast Sensitivity

Although ODE is one of the most common findings in astronauts after LDSF, the clinical impact of SANS-related ODE on functional visual parameters remains ill defined. The development of ODE on Earth may cause decreased color vision, decreased visual acuity, and constriction of the visual field, but to date, no case of SANS has reported to cause permanent visual acuity or visual field loss.^1^ Testing for CS and other tests are currently only performed on the ISS if clinically indicated. Regular CS testing and formal automated visual field testing should be considered as a component of regular vision testing for all astronauts.

Initially termed “Vision Impairment and Intracranial Pressure” (VIIP), SANS was thought to be initially caused by increased ICP.^9^ During spaceflight, there is a loss of hydrostatic pressure, leading to cephalad fluid shifts and possible increased ICP. However, several factors in the clinical presentation and post-flight tests have not been consistent with elevated ICP, leading to a name change from VIIP to SANS. However, direct ICP has never been measured during spaceflight due to the invasiveness of a lumbar puncture procedure, the gold standard for ICP measurements.^1^ Thus, the role of ICP in SANS is still not well understood. As once termed VIIP, idiopathic intracranial pressure (IIH) has been considered to be a terrestrial analog to SANS.^10^ Peripheral CS testing was recently shown to be significantly more affected than central CS in patients with IIH.^11^ Evaluating such patterns during spaceflight with CS may give additional insight into the role of ICP in SANS development.

Numerous theories have been put forward to understand why ICP-related changes might happen, including venous stasis and cerebral edema. In addition, the potential role of the recently discovered ocular glymphatic system has also been postulated in SANS. Some authors have hypothesized that cephalad fluid shifts during LDSF can contribute to elevated ICP. The cerebrospinal fluid (CSF) is predominantly produced in the choroid plexus and then drains into the cerebral venous system. Some studies suggest that a spaceflight-induced reduction in CSF drainage into cerebral veins alongside congestion in this system could result in optic nerve sheath expansion and globe flattening. Although SANS shares some clinical and imaging features with terrestrial IIH, many authors have argued that terrestrial IIH is not a good model for SANS. Typically, IIH presents with clinical features that have rarely, if ever, been described among astronauts with SANS (eg, pulse synchronous tinnitus, transient visual obscurations, and diplopia from sixth nerve palsy).

### Visual Field Perimetry

Automated perimetry serves as a highly useful assessment for various conditions that can affect central vision (eg, macular disruption causing central scotoma) or peripheral vision (eg, glaucoma). There are established risks for visual field loss after LDSF outside of SANS, including increased risk for atherosclerotic disease during spaceflight, radiation exposure, and cardiovascular effects that could theoretically produce visual loss.

Increased intraocular pressure and secondary glaucoma can affect peripheral vision and is one of the most common diseases of irreversible blindness on Earth. While astronauts are less likely to experience glaucoma in year-long missions, current research on goggles as a countermeasure for SANS increases pressure in the orbital area.^12^ Scott, et al concluded in a study on goggles as a countermeasure for SANS that potential compression of the lamina cribrosa may lead to damage of the retinal ganglion cell axons, as observed to terrestrial glaucoma.^12^ Thus, if these future countermeasures are employed for future spaceflight, appropriate visual assessments must also be employed to ensure that peripheral vision is not lost. While traditional perimetry is large and often immobile, head-mounted perimetry is emerging as a useful and accessible method for visual field testing, which will be accessible for spaceflight.^13^

Currently, the gold standard for testing visual field is standard automated perimetry (SAP). Used for diagnosing and monitoring glaucoma, SAP has a patient-interactive approach that helps gather perceptual deficit data in a timely, reproducible, and highly accurate manner – enabling early detection of glaucomatous changes. Some of its limitations, however, include its dependence on patient cooperation, long acquisition times, an uncomfortable posture required from patients, and prohibitive costs of equipment needed. With such limitations, it was important to explore a portable perimetry system – a characteristic of crucial importance when considering its implementation in the ISS.

First described by Hollander, et al in 2000, the Kasha visual system (automated and head-mounted) yielded promising results.^14^ As the headset may be adjusted to fit neatly on the patient’s head, this perimetry device did not need a patient to maintain a specific head position, thus greatly reducing discomfort. The first renders of the virtual reality headset were not widely used due to low screen resolution and small screen sizes; however, as technology advanced, such hardware and software issues were drastically improved, resulting in a highly invaluable testing device.

### Head-Mounted Metamorphopsia Assessment

Amsler grid is onboard the ISS, which helps to identify disruptions in the macula that often manifest as metamorphopsia (distortion) of the central vision. Chorioretinal folds have previously been documented in astronauts and is one of the hallmark findings of SANS. The visual impacts of these folds are dependent on the degree of folding and proximity to the fovea, but can cause metamorphopsia or visual loss. Terrestrially, chronic chorioretinal folds lead to chorioretinal folds-related maculopathy, which leads to a decline in visual acuity.^15^ These significant visual changes could potentially affect an astronaut’s ability to perform critical tasks in LDSF and potentially lead to mission failure. Thus, optimizing the evaluation of such a test is of utmost importance. While the Amsler grid is available onboard the ISS, it is a highly subjective examination and various factors including the surrounding external environment may impact its ability to provide consistent data on any progression of distortion. Even small amounts of distortion detected earlier on can provide further guidance on imaging and possible interventions in the future that are approved for spaceflight.

An essential component of central vision and color perception is the macula. Deficits in macular function can result in severe visual impairment, color detection, and activities of daily living in general such as reading and recognizing faces. Metamorphopsia is a very wide-spread form of visual distortion in several macular diseases (eg, age-related macular degeneration [AMD]). Patients with metamorphopsia often describe fluctuations in vision or deviation of straight lines.

Head-mounted devices have seen a steady rise in their development and application to various terrestrial ocular diseases that include AMD, glaucoma, and amblyopia. Most recently, a study by Ong, et al in 2022 details the development of head-mounted device that could be used as a countermeasure for macular-related visual deficits.^16^ With highly sensitive precision eye-tracking, screen resolution, and wide field view capabilities, head-mounted devices are rapidly becoming an indispensable tool for visual tests.

## Challenges and Future Directions

Although all of the aforementioned functional assessments of vision would be highly useful for astronauts, significant time and space limitations exist on the ISS. The addition of four additional tests of visual function may be a barrier given the current technology onboard the ISS. To address this issue, a head-mounted, multi-modal visual assessment system is currently being built to help detect the subtle visual impacts of SANS.^17,18^ This project is supported by NASA with the goal of developing a non-invasive framework for astronaut vision.^19^

Head-mounted technology has many benefits over traditional forms of visual assessment, as it can use eye-tracking technology, with consistent illumination, be performed rapidly, and with minimal set-up required. These time-savings benefits and ease of use would help allow astronauts to perform this additional visual testing without requiring additional time from their rigorous schedules. Using this head-mounted system, previous research has been able to accurately assess DVA^4^ and assess and mitigate the effects of monocular metamorphopsia from macular disruptions.^16^ This technology may one day be used in vision screening to help regions lacking adequate access to ophthalmic services on Earth.^20^
